# Mist chamber extraction for improved diagnosis of *Meloidogyne* spp. from golf course bermudagrass

**DOI:** 10.21307/jofnem-2020-096

**Published:** 2020-11-06

**Authors:** William T. Crow, Alemayehu Habteweld, Thomas Bean

**Affiliations:** University of Florida Nematode Assay Lab, Entomology and Nematology Department, University of Florida, Gainesville, FL, 32611

**Keywords:** Bermudagrass**, Cynodon, *Meloidogyne graminis*, *Meloidogyne marylandi*, Nematode diagnosis, Nematode extraction, Root-knot, Turfgrass

## Abstract

*Meloidogyne* spp. are among the most damaging plant-parasitic nematodes to golf course bermudagrass in the southern United States. Diagnostic samples processed by centrifugal flotation often recovered only low numbers of vermiform *Meloidogyne* spp. life stages (J2 and males) from soil, while roots were found to be heavily infested by sedentary life stages. Therefore, the University of Florida Nematode Assay Lab (NAL) evaluated mist extraction from turf plugs as a method for diagnosis of *Meloidogyne* spp. from golf course bermudagrass. Soil and turf plugs were obtained from 596 golf course bermudagrass small plots from multiple locations and cultivars over several years, and vermiform *Meloidogyne* spp. extracted from 100 cm^3^ of soil by centrifugal flotation and by mist chamber extraction from four 3.8-cm-diam. turf plugs were compared. Additionally, both extraction methods were performed on 431 golf course bermudagrass diagnostic samples received by the NAL from Florida, 36 golf course bermudagrass diagnostic samples from Texas, and 34 golf course bentgrass/bluegrass samples from California. In the small plots, and the bermudagrass samples from Florida and Texas, mist extraction had higher detection and recovery rates of vermiform *Meloidogyne* spp. than did centrifugal flotation. However, centrifugal flotation had higher detection and recovery rates than mist extraction from bentgrass/bluegrass samples from California. Mist extraction from turf plugs is superior to centrifugal flotation from soil for diagnosis of *Meloidogyne* spp. on golf course bermudagrass, but not on golf course bentgrass and bluegrass.

The grass root-knot nematode, *Meloidogyne graminis* and the Maryland root-knot nematode (*M. marylandi*), have long been recognized as pathogens on turfgrasses (Sledge and Golden, 1964; Grisham et al., 1974; Jepson and Golden, 1987). Recently, these nematodes have increased in importance as pathogens on ultradwarf bermudagrass (*Cynodon dactylon*  ×  *C. transvaalensis*) used on golf greens (Crow, 2018). Non-systematic surveying of root-knot nematodes on turf and forage bermudagrass in Florida (W. T. Crow, unpublished data) have found *M. graminis* to be the most common species, although *M. marylandi* has also been reported (Sekora et al., 2012). These same two species are reported as the predominate *Meloidogyne* spp. associated with golf course bermudagrass in North and South Carolina (Zeng et al., 2012; Ye et al., 2015), Texas (Faske and Starr, 2009), and California and Hawaii (McClure et al., 2012).

A typical turfgrass nematode diagnosis conducted by the University of Florida Nematode Assay Laboratory (NAL) and most other nematode diagnostic laboratories is based on nematodes recovered per volume of soil using centrifugal flotation (Jenkins, 1964) or a similar passive-extraction method. Ideally, turf samples consist of multiple cores collected from the sample area that are mixed to form a composite sample and nematodes are extracted from a standard soil volume of soil. Typically the upper portions of the cores, containing the live turf, thatch and high-organic fraction, are discarded as they add so much debris to the samples that accurate nematode quantification becomes impractical.

During multiple field visits to golf courses with ultradwarf bermudagrass putting greens over the past two decades, chlorotic blotches, often round to oval in shape, that were not associated with known fungal pathogens, were observed by the Sr. author (Fig. 1). These blotches were very similar to those reported on creeping bentgrass greens in the United Kingdom infested with *M. minor* (Karssen et al., 2004). While *Meloidogyne* spp. were suspected as the causal agent of this chlorotic symptom, soil nematode assays often recovered very few *Meloidogyne* J2 although visual inspection of the root systems revealed galls containing many *Meloidogyne* spp. of sedentary life stages. Further, it was noted that infected roots tend to rot off below the galls causing the root systems to be shallow and sometimes roots growing primarily in the thatch layer with very few penetrating into the soil below (Fig. 2).

**Figure 1:**
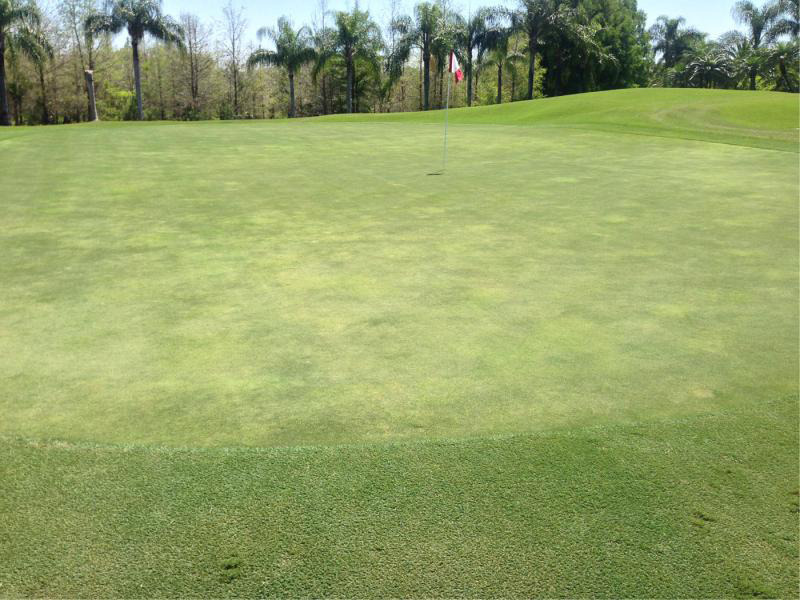
Symptoms of *Meloidogyne graminis* on an ultradwarf bermudagrass golf green. Only low numbers of *M. graminis* vermiform stages were recovered from soil, while staining revealed the roots were infested with large numbers of sedentary stages.

**Figure 2:**
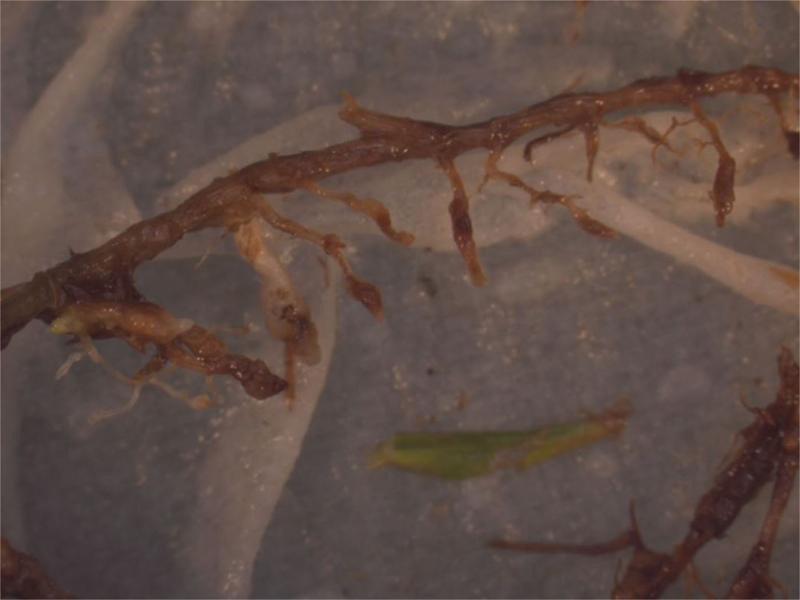
Golf course bermudagrass roots infested by *Meloidogyne graminis* and *M. marylandi* typically rot off just below small galls near the soil-thatch interface, creating an extremely abbreviated root system.

From 2008 through 2015, dozens of turfgrass nematicides trials were conducted at the University of Florida evaluating abamectin for management of sting nematode (*Belonolaimus longicaudatus*). Abamectin greatly increased turf health in many of these trials, while having no measurable effect on sting nematode (Gu and Crow, 2018). In these trials, close observation revealed the turf roots were severely infested by *M. graminis* despite finding few, if any, *M. graminis* J2 in the soil, and those found often were unthrifty in appearance, being burdened by *Pasteuria* spp. attached to their cuticle or by fungal infection.

Laughlin and Williams (1971) reported that throughout the bermudagrass growing season the majority of *M. graminis* J2 recovered from soil extractions were found in the upper 5-cm of soil profile. We hypothesized that the majority of *M. graminis* and *M. marylandi* activity is confined to the extreme upper soil profile and thatch layer where live roots are found, and those J2 found deeper in the soil are often those that are sick and non-infective. This being the case, soil extraction might not provide the most accurate method of diagnosing this nematode or evaluating nematicide efficacy. In the nematicide trials mentioned above, it was hypothesized that the turf visual improvement observed was likely from impacts on *M. graminis* inhabiting the upper profile where abamectin accumulates (Gannon et al., 2017) and not from impacts on sting nematode that predominated deeper in the soil profile.

The NAL set out to develop an effective, efficient, and practical method for extracting *Meloidogyne* spp. vermiform stages for diagnosis on golf course turf, and for evaluation of treatment efficacy in field research. After evaluating several methods, it was determined that a modified Seinhorst (1950) mist method met these criteria. The objective of this research is to compare extraction of *Meloidogyne* spp. vermiform stages using mist extraction from turf plugs with extraction from soil using centrifugal flotation (Jenkins, 1964).

## Materials and methods

### Mist chamber

**Figure 3:**
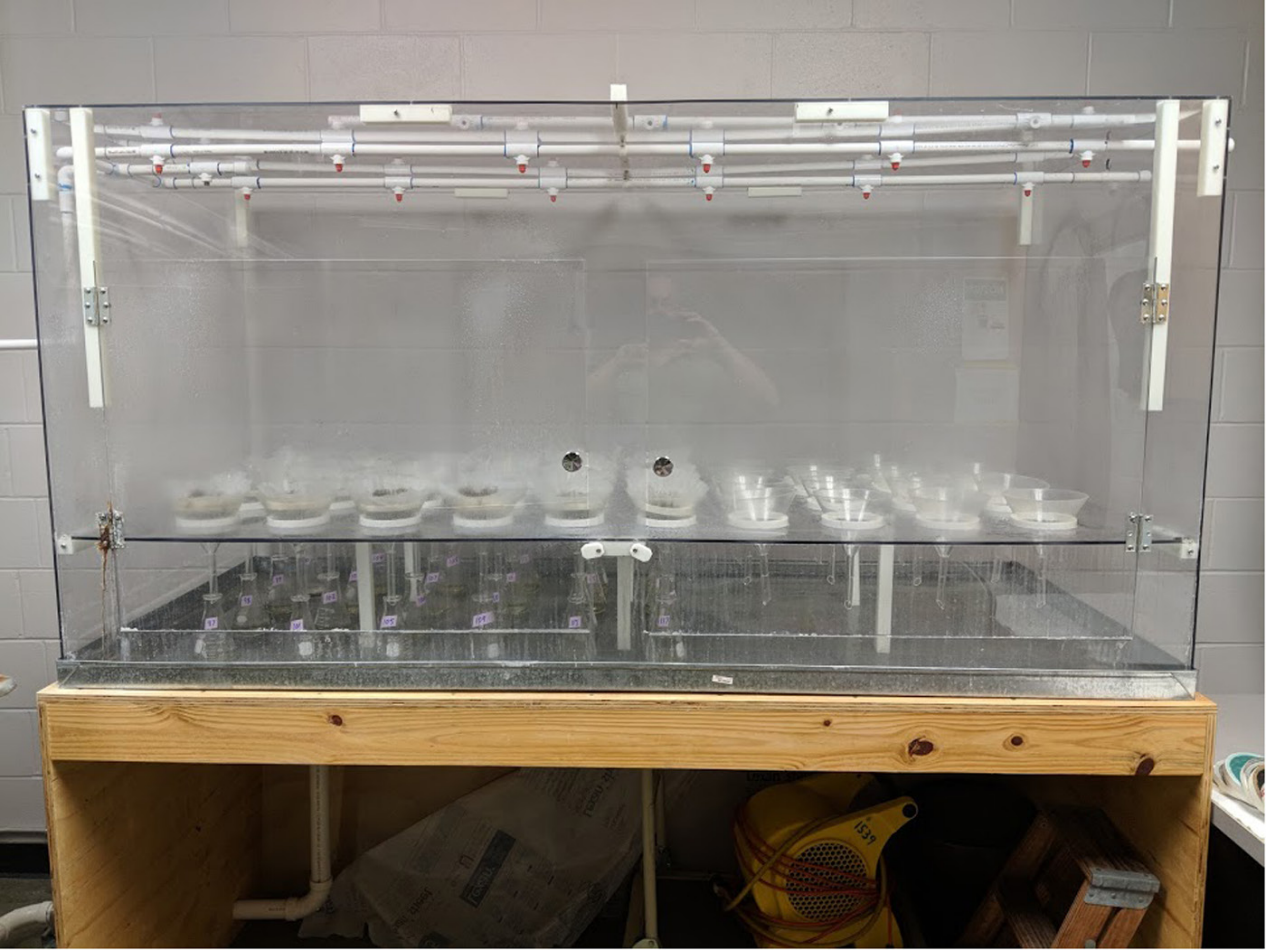
The University of Florida Nematode Assay Lab mist chamber.

**Figure 4:**
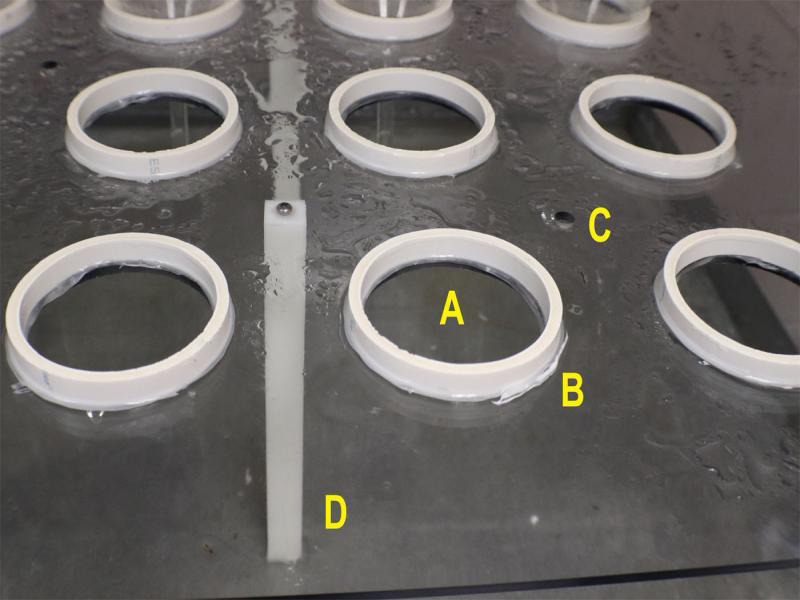
(A) Funnel hole cut in center shelf. (B) Water flow prevention ring. (C) Drain hole. (D) Shelf support.

**Figure 5:**
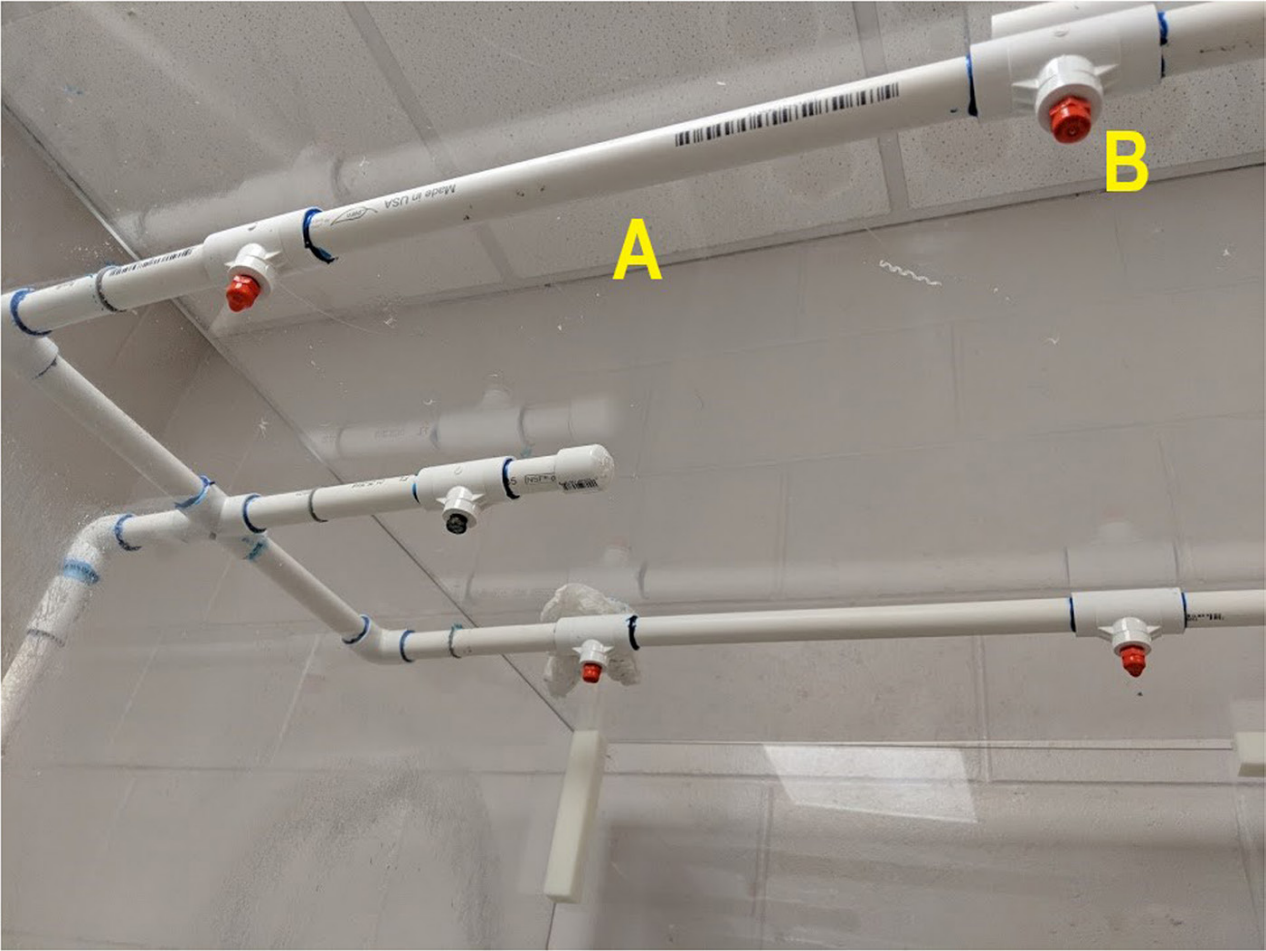
(A) Misting rail. (B) Superfine mist emitter.

The NAL mist chamber (Fig. 3) was built from 0.63-cm-thick polycarbonite sheets that form a chamber 202-cm-wide, 102-cm-deep, and 89-cm-high. The chamber has hinged doors for entry, and a center shelf 29-cm from the bottom of the chamber to hold funnels and samples. The chamber floor is a galvanized metal tray on a wood frame with a drain in the bottom. In total, 40 10-cm-diam. holes were cut into the center shelf at 19-cm centers to hold funnels, and there are four rows with 10 funnel holes each. A 0.63-cm-high ring of 10-cm-diam. PVC pipe was attached to the surface of each hole and sealed with waterproof caulking to prevent water from the shelf from running down the outside of the funnels (Fig. 4). Drain holes (0.63-cm-diam.) were drilled into the shelf to prevent water accumulation on the shelf. Misting rails are 1.27-cm-diam. PVC pipes attached to the ceiling of the chamber running length ways and placed immediately above the spaces between every second funnel row so that drips do not fall into funnels (Fig. 5). Superfine, no drip, low flow mist emitters (DripWorks, Willits CA) were fitted directly into the PVC rails immediately above the gap between each second and third funnel, and at both funnel row ends.

**Figure 6:**
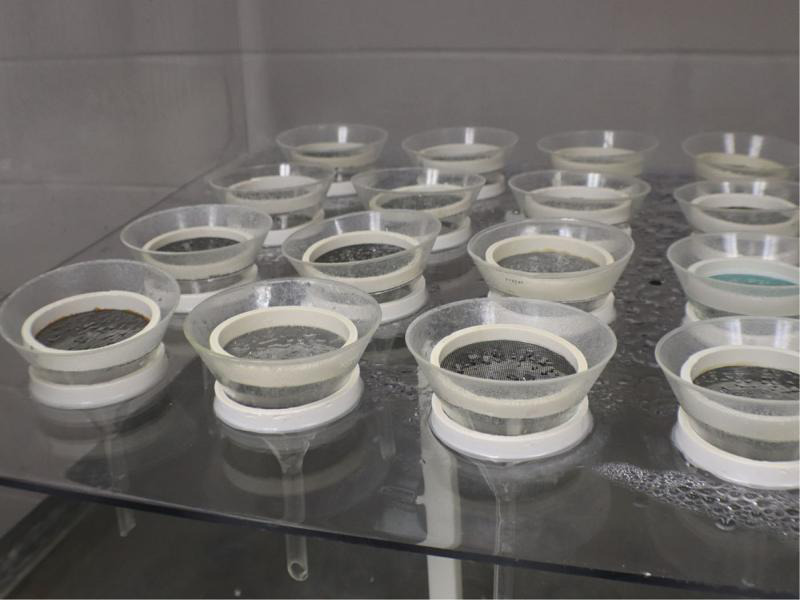
Funnels with sample support rings.

**Figure 7:**
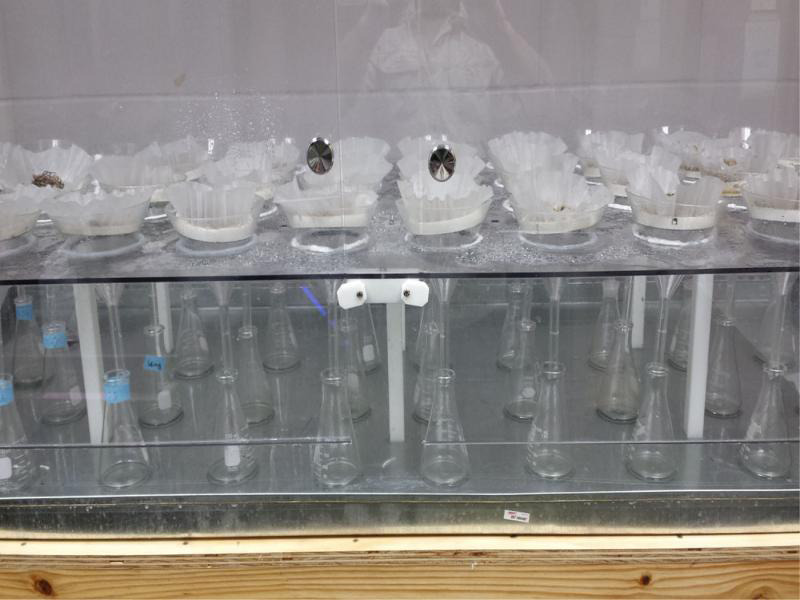
Samples are placed onto coffee filters on the sample support rings. Funnel necks insert slightly into the 250-ml-flaskes.

Glass funnels (15-cm-diam. at top) with 15-cm stems were placed into the funnel holes and 250 ml flasks were inserted under the funnel spouts to collect the emerging nematodes. The stem of the funnels inserted slightly into the flask openings. The water supply was circulated through a sediment filter to remove particulate debris and then into an Autopilot 24-hr recycling timer (Hydrofarm, Petaluma, CA), that regulated the mist length and interval. Sample support rings made of windowscreen glued to 0.63-cm-high and 10-cm-diam. ring, were placed into funnels (Fig. 6), and then a coffee filter was placed onto each screen to hold the sample. Samples were placed directly onto the coffee filter (Fig. 7). Vermiform nematodes emerging from plant material migrated through the filter and then moved via water flow into the flask below. The mist timer was set to apply mist for 10 sec each hour.

### Field plots

**Figure 8:**
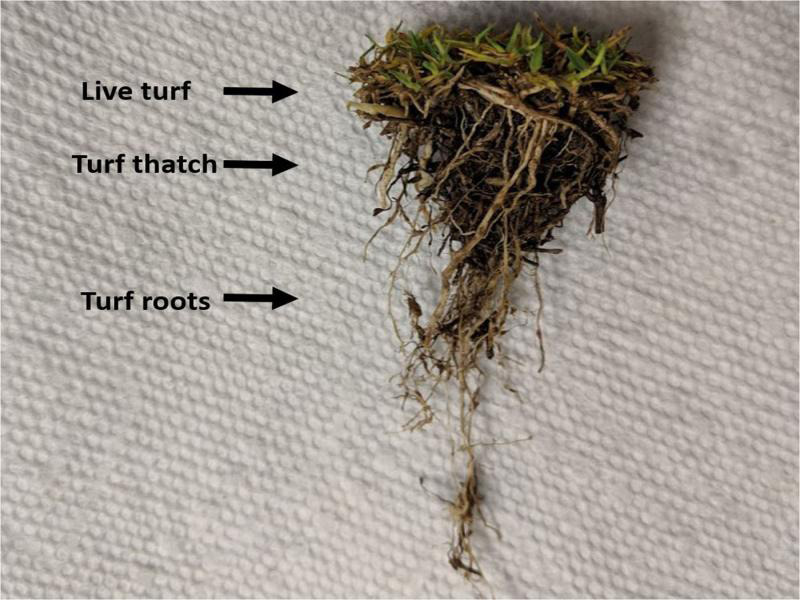
A washed turf plug including live turf, thatch and roots, ready for mist extraction.

Nematode samples for both soil and mist extraction were collected from multiple golf course nematicide trials over multiple years in multiple locations. In all the trial locations, the root-knot nematodes were speciated using mitochondrial DNA haplotyping (Baidoo et al., 2016). The predominant *Meloidogyne* sp. infesting the sites was *M. graminis*, except for the ‘Sunday’ bermudagrass site (Table 1) where it was *M. marylandi*. In this paper only data from the initial, pre-nematicide application, sampling were used. All the trials were conducted on bermudagrass putting greens of varying cultivars and number of plots. Plots were 1.5 m^2^ with 0.61-m borders between adjacent plots. Four cores 6.3-cm-deep were collected from each plot using a 3.8-cm-diam. coring device (Duich Ball Mark Plugger; Turf-Tec International, Tallahassee, FL). Soil was removed from the turf plugs and used for soil extraction, and the plugs, composed of live turf, thatch and roots, were gently washed and used for mist extraction (Fig. 8).

**Table 1.** Comparison of the number of *Meloidogyne* spp. J2 and males recovered from either 100 cm^3^ of soil using centrifugal flotation or from four 3.8-cm-diam. turf plugs using mist extraction.

			J2				Males			
Year	Cultivar	*n*	Soil	Mist	*P* <	*r*^2^	Soil	Mist	*P* <	*R*^2^
2017	Jones Dwarf	77a	6b	436	0.0001c	0.014d	1	40	0.0001	0.059
2017	Tifdwarf	49	225	945	0.0001	0.003	100	184	0.0001	0.085
2017	Tifdwarf	45	92	387	0.0001	0.002	21	92	0.0001	0.150
2017	TifEagle	59	69	368	0.0001	0.048	2	25	0.0001	0.059
2018	Tifdwarf	40	130	466	0.0001	0.005	28	129	0.0001	0.000
2019	TifEagle	71	70	723	0.0001	0.332	22	108	0.0001	0.122
2019	TifEagle	100	24	128	0.0001	0.604	2	13	0.0001	0.374
2019	Sunday	120	14	81	0.0001	0.202	<1	<1	0.0001	0.003
2020	Jones Dwarf	35	22	286	0.0001	0.560	5	26	0.0001	0.244

**Notes:** Samples were collected as the pre-treatment samples for nine bermudagrass golf course nematicide trials on multiple sites, multiple cultivars, and over multiple years. ^a^Number of plots sampled; ^b^Average number of nematodes recovered from *n* samples; ^c^Probability that nematode counts did not differ between methods; ^d^Goodness of fit for regression of nematode counts from mist extraction on the counts from soil extraction.

**Figure 9:**
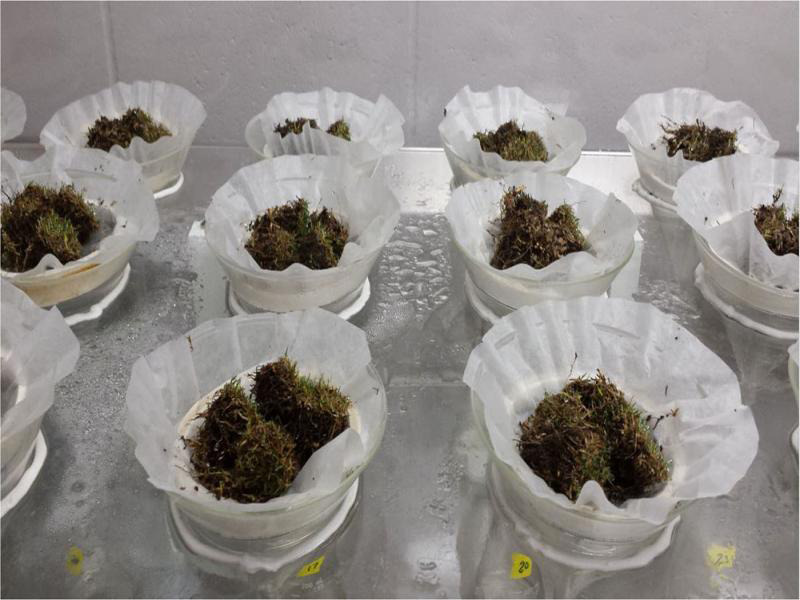
Four washed turf plugs are placed in each funnel in the mist chamber and incubated for 72 hr.

Four plugs from each sample were placed into the mist chamber for 72 hr to extract *Meloidogyne* mobile vermiform life stages (Fig. 9). Because *M. graminis* is a bisexual species, males are abundant and were counted separately from the J2. Vermiform stages of *Meloidogyne* spp. were extracted from 100 cm^3^ of soil by centrifugal flotation. Unlike soil extraction, which was standardized by volume, mist extraction from turf plugs was standardized by turf surface area (four 3.8-cm-diam. plugs yields 34.2 cm^2^ of turf surface area). For each trial the nematodes were counted separately from both extraction methods and subjected to analysis of variance to determine if the counts from the two methods differed from each other. Regression analysis was used to evaluate the relatedness of the counts resulting from the two methods. The nematode counts from mist extraction were regressed on the nematode counts from soil extraction, and the resulting *r*^2^ was recorded.

### Diagnostic samples

The NAL began offering mist extraction of root-knot nematodes from turf plugs as described above an additional diagnostic service in 2016 and began using the NClinic™ (Teaspoon Software, Clarksburg, MD) database for their data management in July 2018. Mist extraction is recommended as a supplement to, and not substitution for, a routine soil assay (Crow, 2018). Often clients request both extraction methods from a single sample. For diagnostic purposes NAL combines the counts of *Meloidogyne* J2 and males and thresholds are based on the total vermiform nematodes recovered per sample. Currently, the NAL uses risk thresholds of 0, 1 ≤ 79, 80 ≤ 299, and ≥ 300 vermiform *Meloidogyne* spp. per 100 cm^3^ of soil or per four 3.8-cm-diam. turf plugs as categories for no, low, moderate, and high risk, respectively, on established golf course bermudagrass.

Florida golf course samples received from July 2018 (when we began tracking samples using the NClinic database) through April 2020, from which both extraction methods were used and from which *Meloidogyne* spp. vermiform stages were recovered, a total of 431 samples, were compared as described for the field plot experiment. Samples with no *Meloidogyne* spp. recovered by either method were not included in the analysis. Additionally, the risk category associated with each sample, based on both extraction methods, were recorded and compared. To evaluate the robustness of the technique, similar analysis was conducted for samples received by the NAL from bermudagrass greens from Texas and bentgrass/bluegrass greens from California with *n*=36 and 34, respectively.

## Results

In all nine field trials, each with 35 to 120 plots per trial, the number of both *Meloidogyne* spp. J2 and males recovered were higher (*P* < 0.0001) from mist extraction of turf plugs than from soil extraction (Table 1). In most cases, the regression of mist extracted nematodes on soil extracted nematodes had a very low (≤ 0.1) *r*^2^, indicating that often there was very little association between the number of vermiform nematodes recovered between the two methods.

**Figure 10:**
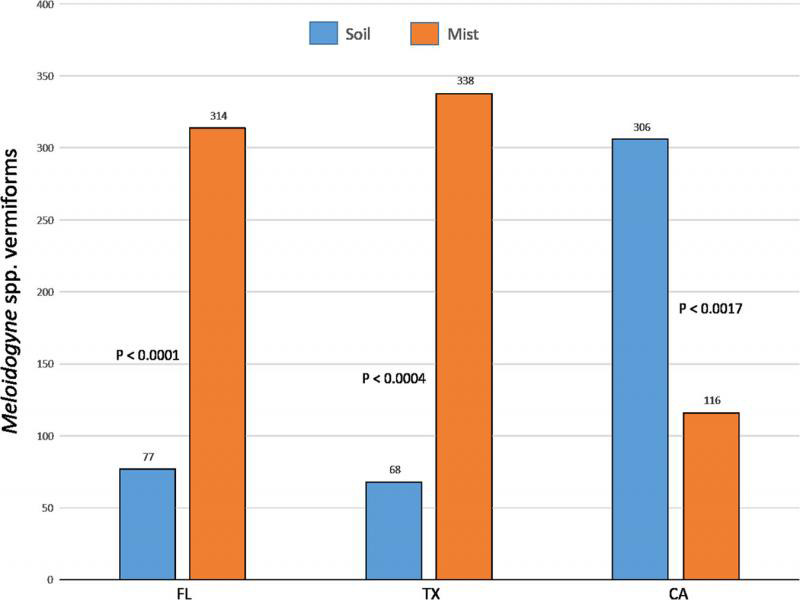
Average number of *Meloidogyne* spp. vermiform stages (J2 and males) recovered from diagnostic samples submitted to the University of Florida Nematode Assay Lab using centrifugal flotation extraction from 100 cm^3^ of soil (Soil) and mist chamber extraction from four 3.8-cm-diam. turf plugs (Mist) from golf course bermudagrasses in Florida (*N* = 431), golf course bermudagrasses in Texas (*N* = 36), or golf course bentgrass/bluegrass in California (*N* = 34).

**Figure 11:**
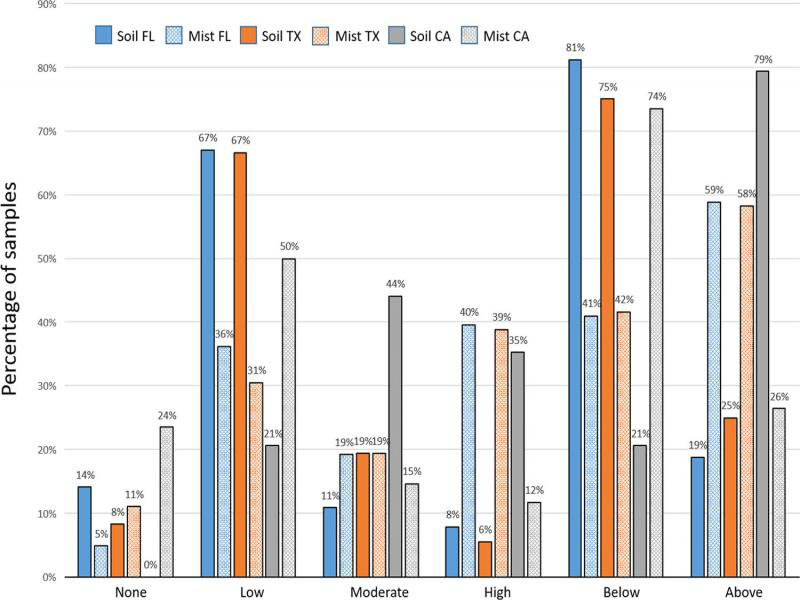
Percentage of samples submitted to the University of Florida Nematode Assay Lab from golf course bermudagrass in Florida (*N* = 431), golf course bermudagrass in Texas (*N* = 36), and golf course bentgrass/bluegrass in California (*N* = 34) that were classified as having no risk, low risk, moderate risk, or high risk of damage from *Meloidogyne* spp. and the percentage of samples that were ‘below threshold’ (no or low risk) and ‘above threshold’ (moderate or high risk) using centrifugal flotation extraction from 100 cm^3^ of soil (Soil) or mist chamber extraction from four 3.8-cm-diam. turf plugs (Mist). Risk thresholds used per 100 cm^3^ of soil or per four 3.8-cm-diam. turf plugs were 0 = no risk, 1 ≤ 79 = low risk, 80 ≤ 299 = moderate risk, ≥ 300 = high risk.

**Figure 12:**
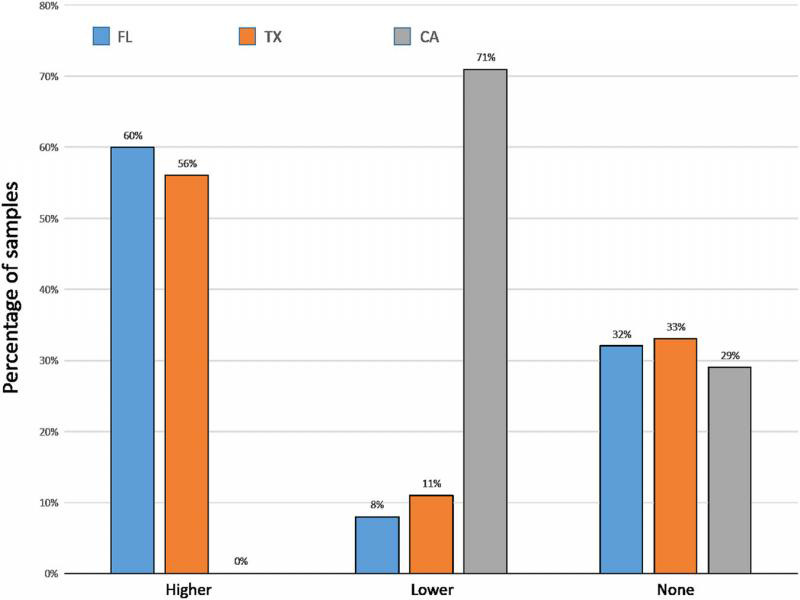
Percentage of samples submitted to the University of Florida Nematode Assay Lab from golf course bermudagrass in Florida (*N* = 431), golf course bermudagrass in Texas (*N* = 36), and golf course bentgrass/bluegrass in California (*N* = 34) from which the resulting risk level was higher, lower, or no different when vermiform *Meloidogyne* spp. were extracted by mist chamber extraction from four 3.8-cm-diam. turf plugs than by centrifugal flotation extraction from 100 cm^3^.

Linear regression of mist extraction counts on soil extraction counts from Florida diagnostic samples yielded a low *r*^2^ of 0.086. From the Florida and Texas diagnostic samples, the average number of vermiform *Meloidogyne* spp. recovered was over four times higher (*P* < 0.0001 and *P* < 0.0004, respectively) from mist extraction than from soil extraction (Fig. 10). The average number of vermiform *Meloidogyne* spp. recovered from California bentgrass/bluegrass samples was almost three times higher from soil extraction than from mist extraction (*P* < 0.0017). When comparing the risk categories resulting from the two extraction methods, mist extraction was more likely to yield numbers ‘above threshold’ (moderate and high risk) than was soil extraction for samples from Florida and Texas, but soil extraction was more likely to yield numbers ‘above threshold’ than mist extraction for California samples (Fig. 11). Soil extraction was almost three times more likely to record a false negative (a count of zero using one method when nematodes were recovered using the other method) than was mist extraction for Florida samples, while for California samples mist extraction resulted in a false negative 25% of the time. In Florida and Texas samples, the resulting risk category from mist extraction was greater than from soil extraction, 60 and 56% of the time, respectively. The resulting risk category in California samples was greater from soil extraction 71% of the time (Fig. 12).

## Discussion

The NAL mist chamber was built for < $1000 in materials. The one described herein was the second one built and had several improvements over the first one. The first chamber was built from acrylic plexiglass sheets, however, we found that polycarbonite sheets experienced less warping, were less brittle, and were less expensive than acrylic plexiglass. The original chamber was built to fit onto a stainless steel sink that was narrower and held fewer samples at one time, having three rows of 11 funnels while the current one has four rows of 10 funnels. One drawback to the current larger chamber is that its increased depth makes it difficult to access the back row of flasks.

In addition to extracting *Meloidogyne* spp., the NAL has found mist extraction from turf plugs to be well suited for extracting certain other genera of plant-parasitic nematodes. The NAL is using this as their standard method to extract *Anguina pacificae*, a species that does not occur on turf in Florida, from California golf course samples. Mist extraction from turf plugs is currently being evaluated for diagnosis of *Hoplolaimus* spp. from golf course bermudagrass in Florida (Crow, 2020). While not currently being used for diagnosis, the NAL staff have observed that mist extraction is likelier to detect *Pratylenchus* spp. than soil extraction by centrifugal flotation. The NAL is also using the mist chamber for diagnostic extraction of endoparasitic nematodes from roots of agronomic and horticultural crops.

Bermudagrass is a warm-season C4 grass and the most common grass used on golf greens in tropic and subtropic climates. Bentgrass and bluegrass are cool season C3 grasses used on golf greens in temperate climates. In Florida, bermudagrass is the most common grass species used on golf courses and cool season grasses are not used at all. In Texas and California, bermudagrass is used predominately in the southern portion of the state and cool season grasses are more commonly used in the northern portion. Most of the Texas golf course samples received by the NAL come from central and southern Texas where bermudagrass is used. However, most of the California golf course samples received originate from northern California on cool season grasses.

The most likely explanation of why the results differed so drastically between bermudagrass greens in Florida and Texas, and bentgrass/bluegrass greens in California is that the predominant *Meloidogyne* species on bermudagrass are likely different and behave differently. Among *Meloidogyne* spp. only *M. graminis* and *M. marylandi* are reported from bermudagrass in Florida (Crow, W. T., unpublished; Sekora et al., 2012) and Texas (Faske and Starr, 2009). Because all the NAL data used herein were from golf course bermudagrass, it is assumed that most of the NAL data shown from those states belong to these two species, but for standard diagnosis nematodes are only identified to the genus level. A survey of golf course turf in the western United States (McClure et al., 2012) found that the predominate species in cool season greens in the continental Pacific states, including California, was *M. naasi*, while *M. graminis* and *M marylandi* were not detected on any of the cool season greens surveyed. Therefore, we assume that *M. graminis* and *M. marylandi* were not frequently detected species in the samples from California.

Different extraction methods are needed for different plant-nematode combinations and mist extraction is likely not the preferred extraction method for *Meloidogyne* spp. diagnosis on all golf greens. While mist extraction is the more accurate extraction method for diagnosis on bermudagrass greens, where *M. graminis* and *M. marylandi* are of primary concern, soil extraction might be more accurate on cool season grasses. A survey of root-knot nematodes from North Carolina (Ye et al., 2015) found that *M. graminis* is common on bermudagrass, *M. nassi* is common on bentgrass, and *M. marylandi* is common on both. Therefore, mist extraction is likely to provide the most accurate diagnosis of *Meloidogyne* on North Carolina bermudagrass, but for North Carolina bentgrass the most accurate method might vary from golf course to golf course based on the predominate species at each location.

In most turf diagnostic situations *Meloidogyne* spp. are one of several genera of plant-parasitic nematodes of concern. For example, on bermudagrass golf greens in the southeastern United States the three genera of major concern are *Belonolaimus*, *Meloidogyne*, and *Hoplolaimus*, while genera of moderate concern are *Trichodorus*, *Mesocriconema*, *Helicotylenchus*, and *Tylenchorhynchus*. Soil extraction provides the most accurate diagnosis for most of these genera. Therefore, in most cases mist extraction from turf plugs should be a supplement to, and not replacement for, standard soil extraction. Poor correlations in *Meloidogyne* spp. vermiforms recovered between the extraction techniques indicates that soil counts cannot simply be subjected to a multiplier to achieve similar results and that mist extraction must be performed as a separate procedure.

Beginning in 2015, the University of Florida Landscape Nematology program began using mist extraction from turf plugs as their standard extraction method for evaluating management of *Meloidogyne* spp. in turfgrass nematicide trials. In previous years it was seldom possible to relate *Meloidogyne* spp. data to turf improvement from nematicide treatments, even when the treatments were believed to be effective. However, since adopting mist extraction we have been very successful correlating counts of *Meloidogyne* spp. vermiform stages recovered with turf health in nematicide trials and these results will be reported in upcoming publications.

The NAL uses 72 hr as their standard incubation time in the mist chamber, realizing that longer incubation time yields even higher nematode recovery. However, in the diagnostic setting both accuracy and speed of diagnosis are important considerations, and 72 hr was selected accordingly. Another consideration for diagnostic labs is robustness of the technique. The NAL receives bermudagrass samples year round, coming from multiple regions with differing environmental conditions even in the same month. For example; during December bermudagrass in northern Florida is largely dormant and nematode activity is low, while at the same time period in southern Florida bermudagrass is actively growing and nematode activity is high. Therefore, our objective is to use methods that are most accurate under varying conditions rather than under specific conditions. These results indicate that mist extraction provides more accurate recovery of *Meloidogyne* spp. from bermudagrass most, but not all, of the time. However, as stated previously we recommend mist extraction be a supplement to, and not replacement of, soil extraction and that submitters have us extract nematodes using both methods.

These results indicate that mist extraction from turf plugs yields improved rate of detection and recovery of *Meloidogyne* spp. on golf course bermudagrass compared to soil extraction by centrifugal flotation. Therefore, this method is a superior extraction method for diagnosis of *Meloidogyne* spp., and evaluation of management practices for these nematodes, on golf course bermudagrass.
